# Functional near-infrared spectroscopy for the detection of fear using parameterized quantum circuits

**DOI:** 10.1038/s41598-025-28942-2

**Published:** 2025-12-29

**Authors:** José L. Gómez-Sirvent, Antonio Fernández-Caballero, Paulo Novais

**Affiliations:** 1 Neurocognition and Emotion Unit, Instituto de Investigación en Informática de Albacete, 02071 Albacete, Spain; 2 Departamento de Sistemas Informánticos, Universidad de Castilla-La Mancha, 02071 Albacete, Spain; 3 Biomedical Research Networking Center in Mental Health (CIBERSAM-ISCIII), 28016 Madrid, Spain; 4 ALGORITMI Research Centre/LASI, Universidade do Minho, 4710-057 Braga, Portugal

**Keywords:** Parameterized quantum circuits, Functional near-infrared spectroscopy (fNIRS), Fear detection, Quantum machine learning, Computational biology and bioinformatics, Neuroscience

## Abstract

Excessive fear in response to certain stimuli may be a key indicator of anxiety disorders. Its detection makes it valuable for the diagnosis and treatment of such pathologies. Quantum computing has shown promising results in processing different types of brain signals. However, its potential for functional near-infrared spectroscopy (fNIRS) signals remains largely unexplored. The present study investigates the application of parameterized quantum circuits (PQCs) for the detection of fear in fNIRS data. To this end, two different quantum architectures and quantum kernels are presented and tested on a publicly available fNIRS dataset. The proposed models are evaluated for subject-dependent and subject-independent classification by cross-validation to measure their performance under different conditions. The cross-validation results showed good performance of the proposed architectures even when trained on a very small dataset. Both analyzed quantum kernels showed high performance as feature extractors. Surprisingly, the subject-dependent approach achieved superior results despite using a training set more than 20 times smaller than that of the subject-independent approach. These results emphasize the power of quantum models in the classification of fNIRS signals and open new avenues for the analysis of this type of brain signals beyond the limitations of classical approaches.

## Introduction

Fear, one of the basic human emotions, arises in response to stimuli that are perceived as an imminent threat, triggering physiological and psychological reactions that prepare the body and mind for fight or flight. Therefore, fear plays a fundamental role in the survival of humans and animals. However, sometimes this emotional response is triggered by stimuli that pose no real threat, interfering with the performance of daily activities and affecting an individual’s well-being. The presence of excessive and persistent fear or anxiety about objects or situations that do not present a real danger is a common symptom in all anxiety disorder^1^. Consequently, techniques for detecting fear or anxiety are a valuable tool in the diagnosis and treatment of this type of disorder^2^.

Various neuroimaging techniques have allowed significant advances in the detection of fear responses as well as the identification of brain areas involved in this process^3^, often referred to as the fear circuit. However, its applicability is limited by factors such as the need for specialized equipment, as in the case of functional magnetic resonance imaging (fMRI), or susceptibility to electromagnetic interference or motion artifacts, as in the case of electroencephalogram (EEG). In this regard, fNIRS provides an efficient alternative to the most commonly used neuroimaging devices. fNIRS is an optical technique that measures variations in near-infrared light absorption to predict changes in oxy- and deoxyhemoglobin concentration associated with activation of different cortical surface areas. The portability and optical nature of fNIRS allows it to be used in conjunction with virtual reality (VR) headsets. This technique has been used in recent years to monitor brain activity in various VR settings. Shi et al.^4^ used fNIRS for stress detection in a virtual industrial environment. Or more recently, Shafiei et al.^5^ successfully employed this technique to improve pain assessment and to explore non-pharmacological pain relief based on VR in cancer patients.

Several relatively standardized preprocessing techniques have been developed to transform raw fNIRS signals into oxy- and deoxyhemoglobin increment signals that can be interpreted by the naked eye^6,7^. However, the human ability to interpret these signals is limited, especially within short time windows. For this reason, various machine learning and deep learning models have been proposed in recent years to analyze this type of signals. Recent literature reviews^8,9^ reflect the growing interest in the application of artificial intelligence in this field. However, these works also highlight as an important limitation the small sample size of most existing fNIRS datasets, especially considering that merging different datasets is usually not feasible due to differences in the specifications and configurations of the devices used. These circumstances pose a challenge to the training of deep learning models, whose generalization ability is often greatly reduced when trained on small datasets. In this context, quantum machine learning (QML) architectures may provide an alternative. Research in this area suggests that these architectures are capable of achieving high performance with few training data^10,11^.

Quantum computing is a new paradigm in information processing. By applying quantum mechanics concepts such as entanglement, superposition, or interference, this emerging discipline seeks to address specific computational challenges with exponentially greater efficiency compared to conventional classical computing^12^. Unlike classical bits, which can only exist in two states, qubits can represent any complex linear superposition of the states $\left| 0 \right\rangle$ and $\left| 1 \right\rangle$, allowing for more efficient information processing. In this context, QML aims to exploit the advantages of quantum computing in machine learning. Any classical bit-representable data, such as text or an image, can be encoded in a Hilbert space for handling in quantum devices. A classical vector of *N* features can be embedded in a quantum space using $Log_2(N)$ qubits^13^.

In QML, logic gates are used to manipulate qubits in much the same way that classical logic gates operate on bits. However, unlike classical gates, some of the quantum logic gates have weights that can be adjusted to manipulate their behavior. PQCs are a fundamental component of most existing QML algorithms^14^, whether they are purely quantum or hybrid classical-quantum. There is a growing interest in the scientific community to explore the synergies between machine learning and quantum computing. In the last decade, numerous studies have introduced quantum circuit-based approaches to create versions of popular machine learning models adapted for quantum computing^15,16^. However, QML is a nascent discipline whose true potential is still unknown. Existing quantum hardware is noisy and has limited computational capacity, which limits the complexity of models. As a result, hybrid models are often used where the quantum component is a small part of the overall architecture^14,17,18^.

Despite the limitations of current quantum devices, the use of quantum circuits and algorithms, either alone or combined with classical machine learning models, has shown to be effective in processing brain signals, especially EEG and fMRI. However, to the best of our knowledge, quantum computing has never been applied to fNIRS signals, except for the use of a quantum annealing algorithm for feature selection^19^. To address this research gap, the present study investigates the feasibility of machine learning models based on PQCs for fear detection in fNIRS signals.

The main contributions of this work are as follows:

- The proposal and implementation of several designs of specialized PQC-based architectures for handling fNIRS signals are introduced.
- The evaluation of the proposed models using a publicly available fNIRS dataset, where each training is repeated several times to ensure a robust evaluation of the proposed architectures, is provided.
- The analysis of the performance of the quantum models in both subject-independent and subject-dependent approaches, allowing a better understanding of their generalization ability with few training samples, is done.

## Related work

This section reviews previous studies on QML for brain signal processing and classical machine learning methods in the context of fNIRS signal analysis.

### QML for the processing of brain signals

Biomedical signals are usually high-dimensional multi-channel data, which poses significant challenges in processing them with quantum algorithms using a small number of qubits. Taha and Taha^20^ proposed a hybrid model for EEG signal classification. They used an autoregressive model as a feature extractor combined with a quantum inspired RNN acting as a classifier. Meanwhile, Li et al.^21^ implemented a framework for EEG feature extraction and classification based on quantum circuits. In this approach, a quantum method was used for both feature extraction, using a quantum wavelet packet transformation, and classification, using a quantum SVM. On the other hand, Lins et al.^22^ used classical preprocessing methods to extract EEG features, which they then used to compare the performance of different hybrid architectures composed of a PQC followed by a MLP as classifier. Their results showed different performance of the studied architectures for different subjects, highlighting the possibility of implementing customized designs for each user. Similarly, Garg, Verma and Singh^23^ used classical feature extraction techniques combined with PCA for dimensionality reduction. They then used an SVM with a quantum annealer and a classical radial basis function kernel for classification, achieving an accuracy comparable to previous proposals based on traditional classical approaches. In the same vein, Saranya and Menaka^24^ used classical feature extraction and selection methods to feed an SVM classifier with a quantum kernel. Their approach slightly outperformed the accuracy obtained with a classical kernel SVM.

In the case of fMRI signals, the ensemble of classical and quantum architectures can increase the performance of the models. Coi et al.^25^ successfully fused the outputs of classical CNNs with 4-qubit PQCs, where amplitude embeddings were used within the PQCs, and combined them in an MLP. They achieved a significant reduction in the number of trainable parameters compared to the ensemble of classical CNNs. On the other hand, Nguyen et al.^26^ explored a fully quantum approach, using a 16-qubit PQC to classify responses to visual stimuli. In this approach, they decided not to use classical feature extraction techniques. By using amplitude embeddings, fMRI signals were efficiently encoded, allowing for an exponential reduction in data dimensionality. The results of this work indicate that amplitude encoding preserves the natural characteristic of fMRI signals. The proposed PQC-based classifier showed higher stability during training and less tendency to overfitting than the classical alternative.

QML has also been shown to be effective when integrated into complex transformer-like models for vision-brain understanding tasks from fMRI. The projection of fMRI data into a Hilbert space can help find connections between fMRI voxels and enhance the semantic information obtained from human perception^27^.

### Machine learning for fNIRS signal analysis

There is strong interest in the scientific community in using machine learning techniques to derive information about mental state from fNIRS beyond what is visually observable in a standard oxy- or deoxyhemoglobin time series plot.

Traditional machine learning methods are widely used in current related research. Their relative simplicity allows efficient generalization on small data sets, making them suitable for fNIRS signal processing. Several recent studies have used support vector machines (SVMs) to classify fNIRS signals. This model has been successfully used to detect abnormal brain activation patterns in adolescents with major depressive disorder^28^, to predict the individual Autism Diagnostic Observation Schedule^29^, or to assess cognitive workload^30^. This classifier has also been shown to be particularly effective when combined with feature reduction techniques such as minimum redundancy maximum relevance^31^ or principal component analysis (PCA)^32^.

Beyond SVMs, several studies have compared the accuracy of various classical machine learning methods in analyzing fNIRS signals. Al-Shargie et al.^33^ compared K-nearest neighbor (KNN), linear discriminant analysis (LDA), naïve Bayes (NB), decision trees (DT), and SVM for assessing brain function after confinement. Their results showed a superior performance of SVM and KNN over the rest of the methods. Similarly, Sanchez-Reolid et al.^34^ analyzed different machine learning classifiers for emotion detection, their results suggest a better performance of tree ensemble-based methods such as bagging trees against KNN. In contrast, Hui et al.^35^ reported a significantly higher accuracy for SVM compared to KNN and random forest (RF) in the early detection of Parkinson’s disease. The discrepancies between the results obtained in different studies suggest that model performance may vary in different fNIRS-based classification tasks, possibly due to the activation of different brain regions depending on the type of stimulus used.

On the other hand, several neural network-based methods have been proposed, which have demonstrated high performance in fNIRS signal processing. For example, Bhutta et al.^36^ proposed three multilayer perceptron (MLP) architectures that showed superior performance in lie detection compared to SVM and KNN algorithms. Yet, most deep learning approaches in this area are based on convolutional neural networks (CNNs) and recurrent neural networks (RNNs)^37,38^. For example, Khan et al.^39^ implemented a lightweight CNN with three inception blocks for the classification of fine anatomical movements. The proposed approach reported superior accuracy to that obtained with LDA and tree ensemble-based models. Fernandez Rojas et al.^40^ developed a hybrid model combining a lightweight CNN with a long short-term memory (LSTM) network, achieving higher accuracy in pain assessment compared to the individual performance of each model. Along the same lines, Jin et al.^41^ proposed a hybrid CNN-Transformer model, which achieved improved performance in emotion recognition. Similarly, Liao et al.^42^ implemented a CNN with Transformer blocks and compared its performance with CNN, LSTM, and Transformer models. Their results showed higher accuracy for the hybrid model, suggesting that combining CNNs for local feature extraction with Transformers for global attention improves the processing of the fNIRS signal.

Beyond these now classical approaches, Kim et al.^19^ applied a quantum annealing algorithm for feature selection. To our knowledge, this is the only case in which a quantum approach has been used to analyze fNIRS signals. The results of their study suggest that quantum algorithms are comparable to classical machine learning algorithms and are feasible for multidimensional neural hemodynamic data analysis.

## Proposed method

### Dataset

A public dataset on height fear detection in VR environments using fNIRS presented by de With, Thammasan and Poel^43^ was used. The dataset includes fNIRS signals from 29 participants exposed to 10 different virtual environments, 5 at ground level and 5 at high height. Of the 29 participants, only 14 reported having a fear of heights. For this reason, and following the approach of the authors of the dataset, only data from these participants were used to test the fear detection models. For each participant, 30 seconds of signal were recorded in each of the 10 environments. However, following the approach of the authors, we only used the time window between 3 and 15 seconds after stimulus application, which is the period in which the hemodynamic response is theoretically expected to appear. The resulting dataset is perfectly balanced, with an equal number of trials labeled as fear and non-fear. For this reason, the authors used classification accuracy as the evaluation metric, and we adopted the same metric to ensure a fair and consistent comparison.

The fNIRS signals were measured at a frequency of 10 Hz with 27 channels, allowing 12 non-overlapping windows of 10 samples for each trial and participant. The signals were pre-processed using the manufacturer’s software of the device used and converted to $\Delta$HbO and $\Delta$HbR values after subtracting the baseline. The authors of the study pointed out that some of the $\Delta$HbR channels were corrupted and had to be excluded, so only the $\Delta$HbO data were used for training and validation of the classifiers. We followed the same approach to ensure a fair comparison of the results.

In a preliminary analysis, most of the data was found to be in a narrow range, but some outliers were detected. These extreme values can be particularly problematic in QML because some of the embeddings used, such as those based on rotations, have periodicity. This can cause very different values to become almost indistinguishable in the quantum feature space before encoding. For this reason, robust scaling was applied to the data before training the models. Robust scaling is an effective scaling technique for dealing with outliers because it scales the data by subtracting the median and dividing by the interquartile range (IQR), which reduces the influence of outliers. In addition, the inverse arctangent was applied to the scaled data to address the problem of outliers:

1 $$\begin{aligned} x_{\text {scaled}} = \text {arctan}\left( \frac{x - Q_2}{Q_3 - Q_1}\right) \end{aligned}$$

where $Q_2$ is the median of the feature values, and $Q_1$ and $Q_3$ represent the first and third quartiles, respectively.

### The QML architectures considered

Two basic architectures were considered for classifying the fNIRS signals. The first, hereafter referred to as AEPQC, processed a single vector composed of all the features from each channel through a single PQC, while the second used a scheme similar to conventional CNNs. This second architecture will be referred to as QCNN.

#### AEPQC

#### The AEPQC architecture

The AEPQC architecture works with one-dimensional vectors, requiring the original signals of 27 channels and 10 features to be flattened into a vector of 270 features. This 1D vector could be encoded by amplitude embeddings using 9 qubits. However, with 9 qubits, it is possible to encode 512 features, which would require adding 242 padding values for each vector. This could be detrimental to the performance of the model. For this reason, we decided to eliminate some channels from the data so that the resulting vectors contained 250 features, which can be encoded with 8 qubits, adding only 6 padding values. To determine which channels could be eliminated with minimal impact on model accuracy, channel selection was based on the Pearson correlation coefficient. The analysis showed that the least informative channels were channels 22 and 24.

**Fig. 1:**
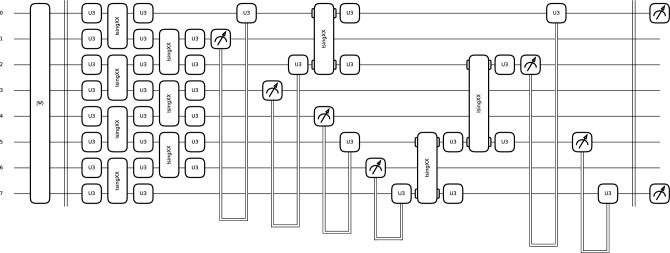
Complex 8-qubit PQC architecture with quantum pooling.

The AEPQC circuit is inspired by the quantum CNN proposed by Cong et al.^44^. In our approach, a single-qubit unitary gate (U3) was used on each wire, followed by IsingXX entanglement gates between pairs of qubits (see Eq. (2) and Eq. (3)). Then another U3 gate was applied to each wire, followed by an IsingXX entanglement gate between the remaining unentangled qubits and another U3 gate. Finally, a quantum pooling operation was performed, using the measurement of alternating neighboring qubits to condition a U3 gate (see Fig. 1).

2 $$\begin{aligned} U3(\theta , \phi , \delta ) = \begin{bmatrix} \cos \left( \frac{\theta }{2}\right) & -e^{i\delta } \sin \left( \frac{\theta }{2}\right) \\ e^{i\phi } \sin \left( \frac{\theta }{2}\right) & e^{i(\delta + \phi )} \cos \left( \frac{\theta }{2}\right) \end{bmatrix} \end{aligned}$$

3 $$\begin{aligned} \text {IsingXX}(\phi ) = \begin{bmatrix} \cos \left( \frac{\phi }{2}\right) & 0 & 0 & -i\sin \left( \frac{\phi }{2}\right) \\ 0 & \cos \left( \frac{\phi }{2}\right) & -i\sin \left( \frac{\phi }{2}\right) & 0 \\ 0 & -i\sin \left( \frac{\phi }{2}\right) & \cos \left( \frac{\phi }{2}\right) & 0 \\ -i\sin \left( \frac{\phi }{2}\right) & 0 & 0 & \cos \left( \frac{\phi }{2}\right) \end{bmatrix} \end{aligned}$$

AEPQC uses only 112 trainable parameters, less than half of what would be needed for an SVM or a single-layer perceptron. However, in QML, and especially when using simulated devices, a small number of parameters is not necessarily associated with low computational cost.

#### The QCNN architecture

**Fig. 2:**
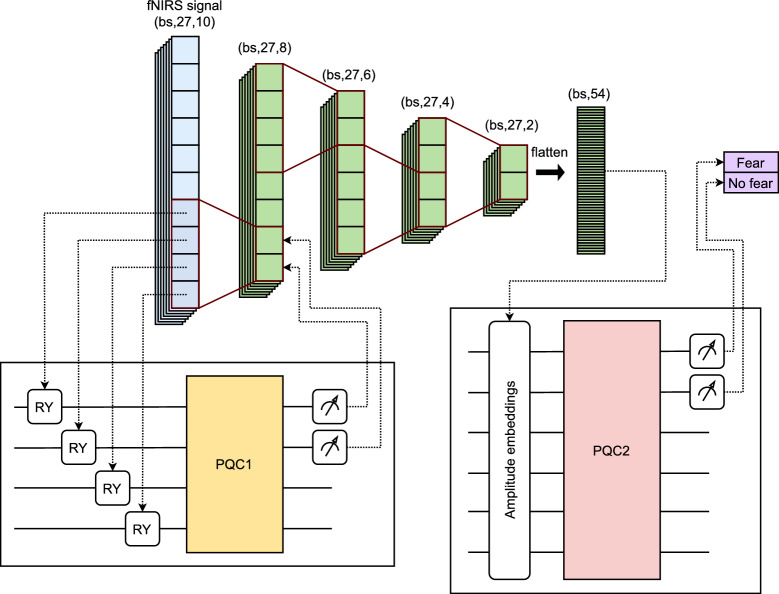
QCNN architecture for fNIRS signal classification (bs refers to batch size).

Figure 2 shows the proposed QCNN architecture. A 4-qubit PQC is used as the convolutional kernel, which traverses the feature space with a step size of 2, achieving a dimensional reduction of 2 features per layer. The network consists of 4 layers, reducing the initial 10 features to only 2. The feature vector is then flattened and encoded by amplitude embedding in a 6-qubit PQC, which acts as the classification head.

**Fig. 3:**
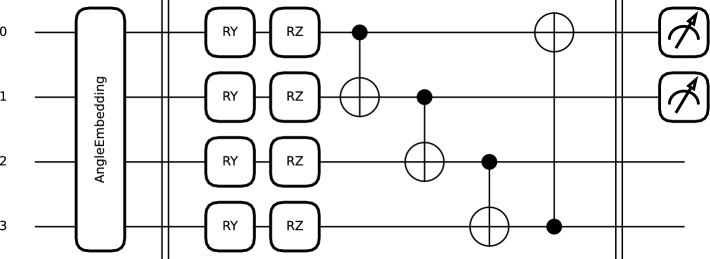
Light quantum circuit (K1) with circular entanglement.

**Fig. 4:**
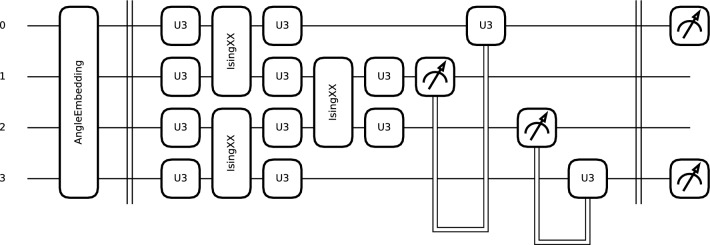
Quantum circuit with quantum pooling (K2).

Two different PQCs were studied to evaluate the impact of the kernel on the final results (see Figs. 3 and  4). In both cases, angle embeddings using RY rotations were used for data encoding (see Eq. (4)). This type of embedding has a [0, 2$\pi$] periodicity. For this reason, the data were scaled to a [0, $\pi$] range before entering the quantum circuit.

4 $$\begin{aligned} RY(\phi ) = \begin{bmatrix} \cos (\phi /2) & -\sin (\phi /2) \\ \sin (\phi /2) & \cos (\phi /2) \end{bmatrix} \end{aligned}$$

The first circuit (K1) was designed to be lightweight, and to avoid distorting the data excessively, it uses only RY and RZ rotations followed by simple circular entanglement with CNOT gates (see Eqs. (4),  (5), and  (6)). Consequently, the circuit used only 2 weights per wire and channel. The output of the circuit was a measurement of the speciation value of Pauli-Z.

5 $$\begin{aligned} RZ(\phi ) = \begin{bmatrix} e^{-i\phi /2} & 0 \\ 0 & e^{i\phi /2} \end{bmatrix} \end{aligned}$$

6 $$\begin{aligned} \text {CNOT} = \begin{bmatrix} 1 & 0 & 0 & 0 \\ 0 & 1 & 0 & 0 \\ 0 & 0 & 0 & 1 \\ 0 & 0 & 1 & 0 \end{bmatrix} \end{aligned}$$

The second PQC (K2) is similar to the one used in the AEPQC architecture (see Fig. 4). The circuit is composed of U3 gates and IsingXX entanglement gates, and as in AEPQC, a dimensional reduction was performed by quantum pooling with U3 gates conditioned on the measurement of neighboring qubits. In this case, however, the gates were distributed so that each wire had the same number of U3 gates. In this circuit, the U3 gates used 9 trainable parameters per wire, and three additional parameters were used for the three entangling gates.

**Fig. 5:**
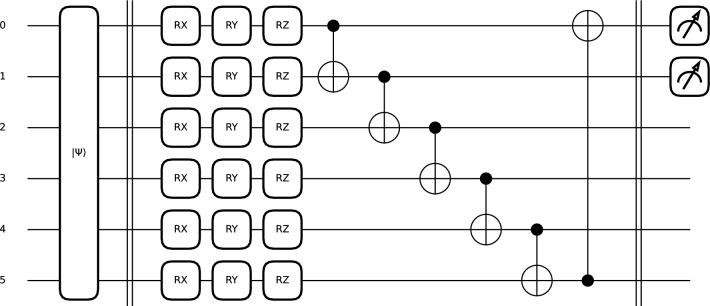
PQC used as a classification head in the QCNN.

A 6-qubit PQC with amplitude embeddings was used as the model classification head (see Fig. 5). An architecture similar to the K1 core was used, except that in this case RX gates (see Eq. (7)) were added. The decision to include these gates was made to increase the expressiveness of the model when handling data encoded with amplitude embeddings.

7 $$\begin{aligned} R_X(\phi ) = \begin{bmatrix} \cos \left( \frac{\phi }{2}\right) & -i\sin \left( \frac{\phi }{2}\right) \\ -i\sin \left( \frac{\phi }{2}\right) & \cos \left( \frac{\phi }{2}\right) \end{bmatrix} \end{aligned}$$

### Training and validation

The architectures were implemented using PennyLane^45^ (version 0.40.0) and PyTorch (version 2.5), utilizing the *default*.*qubit* simulator in PennyLane. The quantum circuits were connected via PyTorch, so the input data was provided as tensors of shape $(batch\_size, num\_channels, temporal\_dimension)$, where $batch\_size$ corresponds to the entire training or validation set, $num\_channels$ = 27 for QCNN and 25 for AEPQC, and $temporal\_dimension$ = 10. The quantum encoding of fNIRS signals–either via amplitude or angle embedding–is performed internally within each model during the forward pass. The same training and validation scheme used by the authors of the fNIRS dataset^43^ was adopted to ensure comparability of results. Both subject-independent and subject-dependent analyses were performed. In the subject-independent approach, a leave-one-subject-out cross-validation was performed. For each iteration, one participant was held out as the validation set, while the remaining participants formed the training set. In the subject-dependent approach, for each subject, each model was trained using the first six trials of the corresponding subject (three fear and three non-fear), and the remaining four trials (two fear and two non-fear) were used for validation.

When working with small validation sets, sometimes minimal variations in model weights translate into large changes in validation accuracy, since it is calculated on the basis of few data. To quantify this variability and better evaluate model performance, we decided to repeat each training and validation process 10 times, initializing the model weights from 10 different random seeds.

The use of simulated quantum devices in classical hardware configurations is inefficient due to the challenges posed by quantum operations, such as entanglement, which complicate parallel processing. In our approach, training was performed using the entire training set at once, as is common in traditional machine learning algorithms, rather than using the mini-batch strategy typical of deep learning. This decision was made because mini-batch processing in the PennyLane simulator becomes less efficient as the batch size decreases.

The Adam optimizer was used to adjust the weights of the models, and the cross-entropy loss was used as the loss function. In a preliminary test by training on the full dataset, it was observed that for learning rates between 0.1 and 0.001, there was a continuous decrease in training loss without instability. For this reason, a learning rate of 0.1 was used in the subject-independent study, and a learning rate of 0.005 was used in the subject-dependent study due to the smaller training set.

An early stopping criterion was set to terminate training after 5 epochs of no improvement in validation loss. However, this criterion was not applied until the training accuracy reached 75%. This decision was made to avoid stopping the training too early in case a small validation loss occurred by chance in the first epochs without the model having really learned.

Accuracy was used as the main metric to evaluate the performance of the models. The size of the validation set (120 samples in subject-independent and 48 samples in subject-dependent analyses) was considered when interpreting the classification results. If the validation set were infinitely large, any model with an accuracy above 50% could be confidently considered better than a random classifier. However, given the small size of the validation set, the accuracy threshold was determined by calculating the minimum accuracy required for the lower bound of the Wilson score interval to exceed 50% at the 95% confidence level:

8 $$\begin{aligned} b_l = \frac{1}{1 + \frac{z^2}{n}} \left( \hat{p} + \frac{z^2}{2n} \right) - \frac{z}{1 + \frac{z^2}{n}} \sqrt{\frac{\hat{p}(1 - \hat{p})}{n} + \frac{z^2}{4n^2}} \end{aligned}$$

where $b_l$ is the lower bound of the Wilson score interval, $\hat{p}$ is the estimated performance, $n$ is the sample size, and $z$ is the critical value of the standard normal distribution for the desired confidence level.

Therefore, a model was considered to perform better than a random classifier if its accuracy exceeded 58.95% in the subject-independent analysis and 64.15% in the subject-dependent analysis.

The complete experimental workflow is summarized as follows:

- Split the dataset according to the subject-independent or subject-dependent strategy.
- Preprocess all trials using robust scaling and inverse arctangent transformation.
- Convert preprocessed trials into PyTorch tensors.
- Encode fNIRS signals internally in the model using amplitude or angle embedding.
- Train the model applying early stopping after 5 epochs without validation improvement and training accuracy > 75%.
- Repeat training and validation 10 times with different random seeds to estimate variability.
- Evaluate performance on the validation set using accuracy, comparing against the Wilson score threshold at 95% confidence.

Finally, to investigate the decision-making process of the proposed models, we performed a channel-wise importance analysis using an occlusion-based method. For each input batch, the baseline output of the model was computed, then each channel was individually masked by setting its values to zero, and the output was recalculated. The difference between the baseline and masked outputs, averaged across the batch and normalized across channels, provided a measure of each channel’s contribution:

9 $$\begin{aligned} \text {Importance}_i = \frac{\text {mean}(|\text {baseline} - \text {masked output}_i|)}{\sum _j \text {mean}(|\text {baseline} - \text {masked output}_j|)} \, , \end{aligned}$$

where $i$ indexes the channels, and $j$ runs over all channels included in the analysis. This analysis was performed for all participants in both subject-independent and subject-dependent evaluations.

## Results and discussion

The proposed QCNN performs a dimensional reduction of the feature space, keeping the number of channels intact, and merges the outputs of all channels using a simple 6-qubit PQC with 18 trainable parameters, acting as classification head (*c_head*). As a consequence of the window size used, the kernel stride and the number of layers, the number of features before entering the classification head coincides with the number of classes. Therefore, a prediction is obtained for each channel and class. Consequently, in this case, it would not be strictly necessary to use the *c_head*, since the predictions from different channels could be fused using simple averaging techniques. In fact, the use of amplitude embeddings in the *c_head* implies a strong transformation of the feature space, which could potentially have a negative impact on the accuracy of the model.

In order to investigate the impact of the *c_head*, a small ablation study was performed using a subject-independent approach, comparing the *c_head* to alternative, simpler feature fusion techniques. Specifically, we evaluated replacing the *c_head* with the average of all channels (*avg*), the maximum value (*max*), or the absolute maximum while preserving sign (*amax*). Table 1 shows the results of the comparison.

**Table 1:** Ablation study comparing classification accuracy (mean and standard deviation) for different feature fusion methods in the QCNN architecture.

Method	K1	K2
c_head	**78.88 (11.54)**	**80.26 (10.64)**
avg	77.64 (13.14)	78.71 (12.98)
max	77.45 (9.78)	79.07 (9.84)
amax	77.17 (10.30)	77.95 (10.01)

Results are reported for both K1 and K2 kernels tested.

Significant values are in bold.

The highest accuracy for either kernel was obtained with the *c_head*, demonstrating the effectiveness of the proposed solution, which allows to exclude that the use of amplitude embeddings excessively distorts the latent feature space, negatively affecting the accuracy of the model. Although the improvement in accuracy obtained with the use of the *c_head* is small, it is important to note that it uses a simple circuit with only 18 trainable parameters.

Table 2 shows the subject-independent classification results of the analyzed models, together with the baseline accuracy established by the authors of the dataset using SVM and LDA. AEPQC shows a high variability in accuracy both between different subjects and across different runs when validated on the same subject. Although it was the model with the highest accuracy in 3 of the subjects, it also had the lowest accuracy in 9 subjects. This high variability suggests that the architecture is prone to overfitting.

**Table 2:** Average classification accuracy (%) and standard deviation (%) for subject-independent cross-validation of the analyzed models.

Subject	LDA^43^	SVM^43^	AEPQC	QCNN-K1	QCNN-K2
1	78.33	78.33	72.50 (2.66)	86.00 (3.01)	**87.67 (2.14)**
2	67.50	66.67	81.75 (11.44)	86.67 (0.88)	**87.50 (0.68)**
3	79.17	80.00	67.83 (3.07)	**82.67 (0.66)**	82.50 (1.04)
4	**84.17**	82.50	52.33 (8.10)	79.67 (2.87)	80.83 (2.83)
5	85.00	85.00	87.42 (2.82)	91.33 (2.87)	**94.17 (2.99)**
6	**72.50**	**72.50**	62.42 (4.72)	70.83 (1.52)	70.83 (3.85)
7	88.33	85.83	36.92 (12.52)	90.83 (2.00)	**91.92 (3.19)**
8	48.33	51.67	47.08 (7.44)	59.50 (4.72)	**62.33 (5.15)**
9	65.83	65.83	65.42 (6.54)	81.50 (2.54)	**81.58 (3.00)**
10	87.50	85.83	63.75 (4.77)	93.08 (2.36)	**94.33 (1.51)**
11	53.33	55.83	**82.58 (8.88)**	67.92 (7.86)	72.83 (8.00)
12	68.52	67.59	57.78 (11.42)	68.24 (2.39)	**68.61 (3.89)**
13	69.17	68.33	**87.66 (1.56)**	86.42 (2.42)	83.08 (6.05)
14	57.50	57.50	**91.83 (2.93)**	59.67 (2.49)	65.42 (4.43)
Mean	71.80 (12.75)	71.67 (11.60)	68.38 (16.55)	78.88 (11.54)	**80.26 (10.64)**

Significant values are in bold.

Overfitting is usually associated with models that have a very large number of parameters. However, it is also common in simpler models, such as decision trees. Despite having only 112 trainable parameters, AEPQC shows instability in validation that was not observed during training, which could be an indicator of overfitting. The overall accuracy of AEPQC in subject-independent classification is lower than the baseline established with LDA and SVM. However, in 10 of the 14 subjects, the accuracy was above 59%, the threshold above which the model was considered to perform better than a random classifier.

Furthermore, the QCNN architecture achieved significantly higher and more stable classification results compared to AEPQC. Although there are no major differences between the two kernels tested, the K2 kernel showed slightly better performance. The QCNN outperformed the baseline in 12 out of the 14 subjects and reported an accuracy higher than 59% in all subjects, demonstrating the model’s effectiveness and indicating that the results are statistically meaningful rather than due to random chance. The overall cross-validation accuracy was 78.88% with kernel K1 and 80.26% with kernel K2, significantly higher than the 71.80% reported by the LDA baseline.

**Fig. 6:**
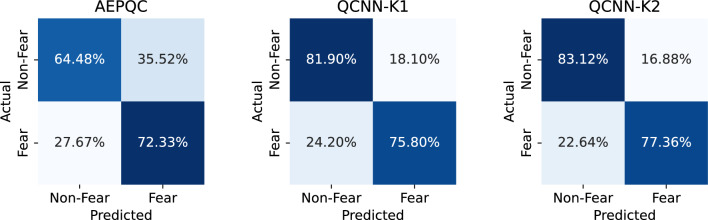
Normalized confusion matrices for the subject-independent evaluation of the analyzed models.

Figure 6 shows the normalized confusion matrices for the subject-independent evaluation of the analyzed models. The matrices illustrate the distribution of true positives, true negatives, false positives, and false negatives across all participants. Overall, all models achieve a high proportion of correct classifications, demonstrating effective discrimination between fear and non-fear trials. The AEPQC model shows slightly higher misclassification of non-fear trials, whereas the QCNN models tend to classify non-fear trials more accurately than fear trials.

**Fig. 7:**
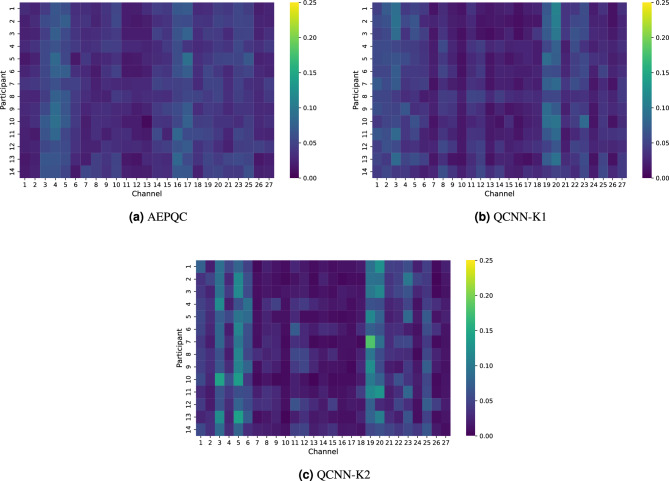
Channel-wise importance of fNIRS signals in subject-independent evaluation.

Figure 7 shows the channel-wise importance of fNIRS signals for each participant in the subject-independent evaluation. For the simplest model, AEPQC, the attention is relatively homogeneous across channels. In contrast, the QCNN architectures, and particularly QCNN-K2, which is the most complex, show a more concentrated attention on specific channels. This suggests that as model complexity increases, the networks tend to rely more heavily on a subset of informative channels to make their predictions, reflecting a more specialized feature extraction process.

On the other hand, the proposed model architectures have also been tested using a subject-dependent approach. This type of analysis may be simpler because the model only needs to learn patterns from a single subject, rather than generalizing across multiple individuals with different physiological responses. However, it poses a significant challenge because the training set is more than 20 times smaller than in the subject-independent approach. Table 3 shows the subject-dependent cross-validation results of the proposed architectures.

**Table 3:** Average classification accuracy (%) and standard deviation (%) for subject-dependent cross-validation of the analyzed models.

Subject	LDA^43^	SVM^43^	AEPQC	QCNN-K1	QCNN-K2
1	41.67	56.25	**90.42 (3.43)**	76.67 (6.86)	82.50 (4.41)
2	52.08	54.17	**92.29 (1.01)**	83.54 (3.32)	86.25 (3.70)
3	58.33	64.58	**69.58 (6.89)**	51.88 (6.91)	54.38 (7.70)
4	85.42	85.42	83.54 (11.60)	85.21 (2.30)	**86.04 (1.98)**
5	89.58	83.33	**100.00 (0.00)**	97.50 (2.37)	97.50 (3.07)
6	58.53	58.33	**79.38 (7.11)**	65.00 (5.36)	62.29 (4.33)
7	97.92	**100.00**	**100.00 (0.00)**	**100.00 (0.00)**	**100.00 (0.00)**
8	**81.25**	**81.25**	79.38 (3.17)	37.50 (7.28)	37.29 (15.44)
9	45.83	45.83	68.96 (16.27)	94.17 (3.51)	**96.67 (1.08)**
10	85.42	83.33	63.54 (3.14)	88.96 (3.93)	**90.63 (3.71)**
11	64.58	60.42	88.54 (2.46)	86.04 (4.61)	**89.58 (2.78)**
12	75.00	**77.78**	72.22 (12.35)	68.06 (6.96)	67.22 (6.38)
13	62.50	60.42	**100.00 (0.00)**	95.63 (3.17)	98.13 (2.49)
14	62.50	58.33	**90.63 (1.10)**	86.67 (7.36)	85.00 (10.10)
Mean	68.60 (17.29)	69.25 (15.64)	**84.18 (12.33)**	79.77 (18.24)	80.96 (18.75)

Significant values are in bold.

The overall accuracy of the baseline models was below that obtained in the subject-independent analysis. In half of the subjects, accuracy was below the 64.15% threshold of the random classifier. Nevertheless, the proposed quantum models improved their average accuracy with respect to the subject-independent approach. For QCNNN, the improvement was minimal and accompanied by an increase in the standard deviation. In contrast, AEPQC showed a substantial improvement of more than 16 percentage points, going from being the worst performing model in the subject-independent approach to being the classifier with the highest accuracy and lowest standard deviation in the subject-dependent approach.

**Fig. 8:**
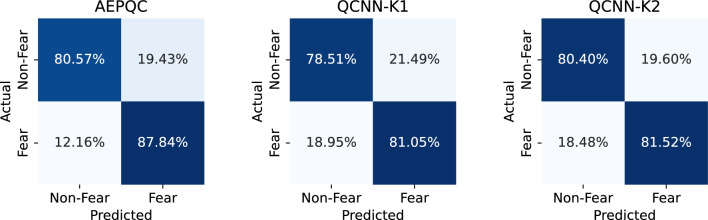
Normalized confusion matrices for the subject-dependent evaluation of the analyzed models.

Figure 8 presents the normalized confusion matrices for the subject-dependent evaluation of the analyzed models. The results indicate that all models correctly classify the majority of trials, demonstrating effective discrimination between fear and non-fear conditions. The AEPQC model shows a tendency to misclassify non-fear trials slightly more often than fear trials, while the other two models achieve a more balanced performance across both classes.

**Fig. 9:**
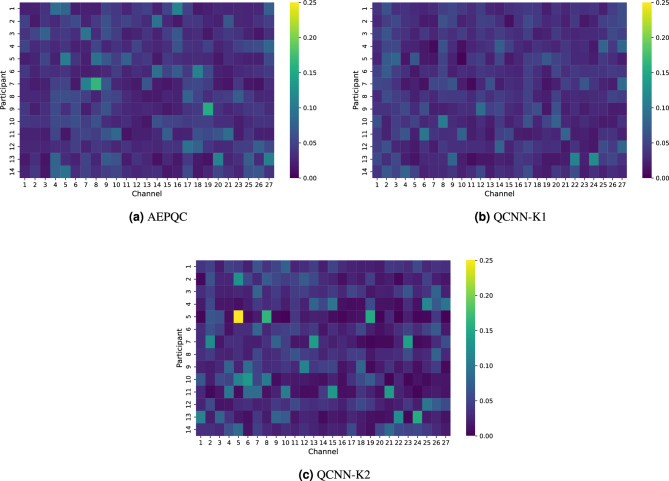
Channel-wise importance of fNIRS signals in subject-dependent evaluation.

Figure 9 shows the channel-wise importance of fNIRS signals for each participant in the subject-dependent evaluation. Unlike the subject-independent approach, where attention was consistently concentrated on specific channels across participants, here the distribution of attention varies considerably between subjects. This variability can be attributed to differences in the quality of the fNIRS signals recorded from each participant, which depend on factors such as the placement of the optodes and potential movement during the experiment. As a result, each model adapts its focus to the channels providing the most reliable information for the individual subject, which could explain the higher accuracy observed in the subject-dependent evaluation.

An additional aspect highlighting the practical viability of the proposed models is their training efficiency. All models were trained using the PennyLane simulator on a standard Kaggle environment with a dual-core Intel Xeon processor, without requiring specialized hardware. In this setup, AEPQC achieves approximately 18 training epochs per minute, while QCNN-K1 and QCNN-K2 reach around 10 and 5 epochs per minute, respectively. The training set in this evaluation consisted of 1560 samples. These rates are comparable to classical machine learning models such as SVM, demonstrating that PQC-based architectures can be trained efficiently even on modest computational resources. Notably, the AEPQC model, despite having roughly half the number of trainable parameters compared to the equivalent SVM model, achieves excellent performance, particularly in the subject-dependent evaluation. This underscores the effectiveness of lightweight, problem-specific simulated quantum circuits in achieving high accuracy with reduced model complexity, offering a practical alternative to classical models for fNIRS-based fear detection.

These results are consistent with previous research suggesting that quantum models can achieve high accuracy even when trained with very few samples^10,11^. Furthermore, they demonstrate the effectiveness of amplitude embeddings in drastically reducing the dimensionality of signals while preserving relevant information. Nguyen et al.^26^ successfully applied a simple PQC with amplitude embeddings to fMRI signal processing. Our results confirm that this type of encoding is also effective for fNIRS signals.

On the other hand, the QCNN reported results above 64.15% in most subjects. However, very poor results were obtained in a few subjects. Specifically, subject 8 had an accuracy significantly below 50%. This high variability suggests that QCNN may be too complex for the size of the training set used in the subject dependent approach. In this regard, different solutions could be proposed to deal with the overfitting problem. The use of data augmentation techniques has been shown to be effective in many classical machine learning problems^46–48^. However, there is no clear evidence of its effectiveness in QML. Zhouli, Wang and Parampalli^49^ compared the impact of different levels of data augmentation on a QCNN and a classical CNN. Their results showed no improvement in the performance of the QCNN when data augmentation was introduced, unlike the classical CNN.

Within the QCNN, using the K2 kernel resulted in an improvement of just over one percentage point over K1. However, K1 has a significantly lower complexity than K2, making it a more suitable choice for integration into complex networks, despite its slightly lower performance as a feature extractor.

In our study, the same architectures were used for both subject-dependent and subject-independent analyses. Our results showed that despite working with the same signals, the type of analysis, and therefore the size of the training dataset, significantly influenced the performance of the models. Transfer learning approaches have shown effectiveness in QML^50,51^. However, their applicability to fNIRS signal processing is limited considering the heterogeneity of existing fNIRS datasets. Therefore, the use of lightweight problem-specific architectures emerges as a potential solution to the limitations of data augmentation and transfer learning in this area.

In large classical-quantum hybrid models, it is possible to use random quantum circuits as kernels^52–54^. However, in small fully quantum models, circuit design becomes critical. Given the need for problem-specific models, quantum architecture search algorithms^55,56^ could be very useful to find the optimal design for each task or even for each subject. As in classical logic circuits, quantum circuits can also benefit from simplification methods to achieve more efficient solutions. Methods such as ZX-calculus have proven effective in reducing circuit size by optimizing gate layout and eliminating redundant operations^57^.

### Limitations

This paper demonstrates the potential of QML for the classification of fNIRS signals. However, it is important to note the limitations of the study conducted.

The evaluation of the architectures on a simulator rather than on real quantum hardware is an important limitation. The results obtained in this experiment are likely to be superior to those that would be obtained on a real quantum computer, since they are not affected by the noise present in current quantum devices. However, given the advances in quantum hardware development, it is reasonable to expect that less noisy devices will be introduced in the coming years, which could lead to results closer to those obtained in simulators.

The use of a small dataset is another limitation, as it makes it difficult to generalize the results. However, it is important to note the problem-specific nature of this type of dataset. The architectures presented in this study were designed taking into account the sampling rate and number of channels of the fNIRS device used. Given the variability in fNIRS hardware and optode configurations, and the prevalence of subject-dependent scenarios, working with limited data is often an inherent and expected condition in this field. However, to address the challenge posed by the small dataset size, the Wilson confidence interval was employed to assess whether the reported accuracies are statistically meaningful and unlikely to have occurred by chance, thus providing a more reliable validation despite the limited data.

Finally, using a classical optimizer introduces another limitation. Adjusting the weights with a non-quantum-aware algorithm may lead to solutions that do not exploit the full potential of quantum models. However, the use of classical optimizers in quantum models is a widely used and accepted approach. Although quantum optimization approaches could provide better alignment with the underlying structure, this remains a largely unexplored area. Consequently, their effectiveness is uncertain and could lead to unpredictable behavior.

## Conclusions and future work

In the present work, we explored the use of PQCs for fear detection in fNIRS signals. To the best of our knowledge, this is the first case of applying a quantum classifier to this type of signals. Two architectures with different levels of complexity were presented and tested for both subject-dependent and subject-independent classification in cross-validation. The results showed good performance of the QCNN architecture in both approaches. However, the AEPQC exhibited inconsistent performance, achieving high accuracy in the subject-dependent test but significantly lower accuracy in the subject-independent scenario.

These results highlight the effectiveness of the proposed models for fNIRS signal classification with limited training data. Nevertheless, the observed variability, especially in the subject-dependent approach, highlights the importance of adjusting the model complexity to the size of the available training set.

This research is intended to be a first step in an underexplored area and to pave the way for future research. Further work could focus on refining quantum architectures, exploring alternative optimization strategies, or investigating techniques to improve the generalization of the models. In addition, evaluation of the proposed models on real quantum hardware could help to better understand their practical feasibility and performance under real-world conditions.

## Data Availability

The dataset used in this study is publicly available at https://data.4tu.nl/articles/_/17302865. The source code developed for this research is available from the corresponding author upon reasonable request.
